# RNAi in Arthropods: Insight into the Machinery and Applications for Understanding the Pathogen-Vector Interface

**DOI:** 10.3390/genes3040702

**Published:** 2012-11-06

**Authors:** Annette-Christi Barnard, Ard M. Nijhof, Wilma Fick, Christian Stutzer, Christine Maritz-Olivier

**Affiliations:** 1 Department of Biochemistry, University of Pretoria, Pretoria, 0002, South Africa; E-Mails: annettechristi@gmail.com (A.C.B.); christian.stutzer@gmail.com (C.S.); 2 Institut für Parasitologie und Tropenveterinärmedizin, Freie Universität Berlin, Königsweg 67, 14163, Berlin, Germany; E-Mail: ardmenzo.nijhof@fu-berlin.de; 3 Department of Genetics, University of Pretoria, Pretoria, 0002, South Africa; E-Mail: wilma.fick@up.ac.za

**Keywords:** RNA interference, vector, disease, mosquito, ixodid ticks, body louse, kissing bug, tsetse fly, transgenesis, vaccine, drug target

## Abstract

The availability of genome sequencing data in combination with knowledge of expressed genes via transcriptome and proteome data has greatly advanced our understanding of arthropod vectors of disease. Not only have we gained insight into vector biology, but also into their respective vector-pathogen interactions. By combining the strengths of postgenomic databases and reverse genetic approaches such as RNAi, the numbers of available drug and vaccine targets, as well as number of transgenes for subsequent transgenic or paratransgenic approaches, have expanded. These are now paving the way for in-field control strategies of vectors and their pathogens. Basic scientific questions, such as understanding the basic components of the vector RNAi machinery, is vital, as this allows for the transfer of basic RNAi machinery components into RNAi-deficient vectors, thereby expanding the genetic toolbox of these RNAi-deficient vectors and pathogens. In this review, we focus on the current knowledge of arthropod vector RNAi machinery and the impact of RNAi on understanding vector biology and vector-pathogen interactions for which vector genomic data is available on VectorBase.

## 1. Introduction

Vector-borne diseases are caused by pathogens that are transmitted between vertebrate hosts by another organism, predominantly via the saliva of blood-sucking arthropods, such as mosquitoes, fleas, lice, biting flies, mites and ticks. These may cause high incidences of morbidity, and in some instances mortality, not only affect low-income developing countries but also developed countries [1,2]. Biophysical, anthropogenic and climate changes affect vector population dynamics and disease transmission, as they influence the survival and reproduction rates of vectors. This in turn influences the distribution and abundance, habitat suitability, intensity and temporal pattern of vector activity throughout the year, and rates of development, survival and reproduction of pathogens within vectors [3]. As the severity of any vector-borne disease depends on the pathogen, the invertebrate vector and the human or animal host, understanding of the host-vector-pathogen interfaces is vital to gain insight into vector biology as well as pathogen survival and transmission.

In this post-genomic era, scientists aim to obtain an integrative view of the host-vector-pathogen interfaces using studies that comprise genomics, transcriptome and proteome analyses, population biology and vector biology. In combination, this will expedite the development of novel therapeutics and vaccination alternatives and is already a major focus in research on mosquitoes [4], trypanosomes [5], tick-borne pathogens [6,7] as well as species-specific insecticides [8]. In 2006, VectorBase, a NIAID-funded Bioinformatic Resource Center, focused on invertebrate vectors of disease, was established online [9]. To date, it houses the genomes of *Anopheles gambiae* (vector for malaria), *Aedes aegypti* (vector for yellow fever and dengue fever), *Culex pipiens* (vector for lymphatic filariasis and West Nile fever), the body louse *Pediculus humanus* (vector for epidemic typhus), the triatomine *Rhodnius prolixus* (vector for Chagas disease), the tsetse fly *Glossina morsitans* (vector for sleeping sickness) and the tick *Ixodes scapularis* (vector for Lyme disease) (http://www.vectorbase.org). The availability of these vectors’ genomic resources, in combination with completed genome sequences of a number of their associated pathogens (Table 1), has necessitated the development of annotation/ontology tools to describe vector processes that are involved in vector survival and disease transmission. As a result, researchers are now faced with annotation of a large number of genes with unknown functions [10]. 

RNA interference, which refers to the silencing of the expression of a single gene, coupled with the resulting phenotype, has been used with great success in most of the abovementioned vectors for a growing understanding of function, as described in Section 2. The molecular mechanisms underlying the processes of dsRNA uptake, silencing, amplification and spreading of the silencing effect, as well as the proteins involved in these processes, however, remain to be clarified (Table 2). Based on RNAi methodology, the development of high-throughput genome-wide assay platforms for disease vectors is rapidly providing insight into host genes that are affected during pathogen infection. RNAi studies offers to facilitate gene annotation, unraveling the molecular interactions between vector-host and vector-pathogen and ultimately aiding in the discovery of novel vaccine and drug targets.

In this review, we bring together the current knowledge on the RNAi machinery and mechanisms in vectors housed on VectorBase. In the first part, we focus on the progress made in terms of expanding our knowledge on invertebrate RNAi machinery and processes, followed by examples were RNAi has expanded our knowledge of the host-vector-pathogen interfaces. Finally, we highlight the current status and future directions of RNAi and ‘omics technology in controlling vectors and their associated pathogens.

**Table 1:** Summary of vectors addressed in this review with their respective diseases, hosts and genomic summaries. Adapted from [11,12].

Vector	Pathogen	Disease	Gene counts
*Culex quinquefasciatus* (Southern house mosquito)	West Nile virus *Wuchereria bancrofti*	West Nile Fever Lymphatic filariasis	Genome (bp): 539,959,374 Known protein-coding genes: 18,858 Gene exons: 75,303 Gene transcripts: 23,049
*Anopheles gambiae*	Plasmodium spp.	Malaria	Genome (bp): 278,253,050 Known protein-coding genes: 12,670 Gene exons: 56,210 Gene transcripts: 14,974
*Aedes aegypti* (Yellow fever mosquito)	Dengue virus Chikungunya virus (CHIKV) Yellow fever virus	Dengue fever Chikungunya Yellow fever	Genome (bp): 1,310,090,344 Known protein-coding genes: 15,704 Gene exons: 66,827 Gene transcripts: 18,769
*Ixodes scapularis* (Deer- or blacklegged tick)	*Borrelia burgdorferi* *Borrelia miyamotoi*	Lyme Disease Relapsing fever	Base Pairs: 1,388,472,180 Novel protein-coding genes: 20,457 Gene exons: 93,988 Gene transcripts: 24,925
*Pediculus humanus corporis* (Body louse)	Infestation of lice *Rickettsia prowazekii* *Bartonella Quintana* *Borrelia recurrentis*	Pediculosis Epidemic typhus Trench fever, endocarditis Louse-borne relapsing fever	Base Pairs: 108,367,968 Known protein-coding genes: 10,773 Gene exons: 69,506 Gene transcripts: 10,994
*Rhodnius prolixus* (Kissing Bug)	*Trypanosoma cruzi*	Chagas Disease	Base Pairs: 561,474,548 Pseudogenes: 1,148 Gene exons: 184,075 Gene transcripts: 36,307
*Glossina morsitans* (Tsetse fly)	*Trypanosoma brucei* *Trypanosoma b. brucei*	African trypanosomiasis (sleeping sickness) Animal trypanosomiasis (Nagana)	Base Pairs: 363,107,930 Gene exons: 64,464 Gene transcripts: 12,362

## 2. Invertebrate RNAi Machinery

**Figure 1:**
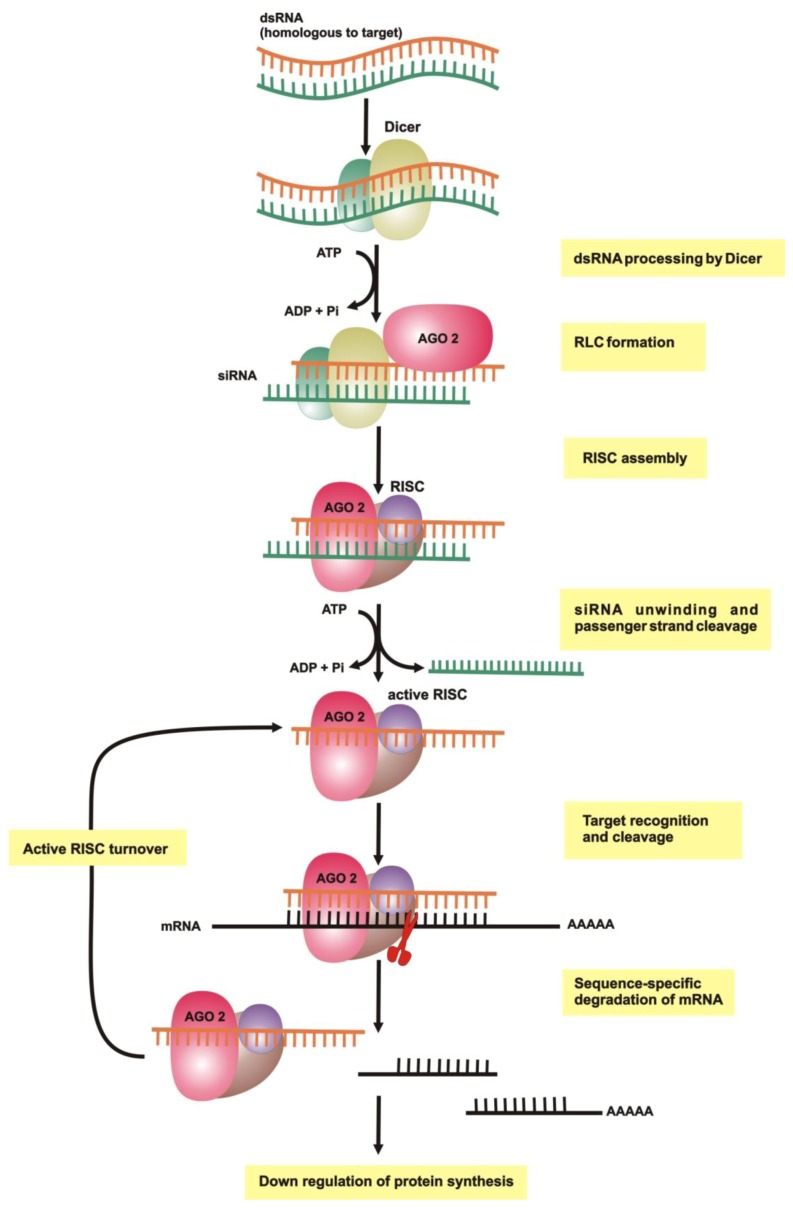
The RNAi process and biochemical machinery involved. Adapted from [30,31]. dsRNA is processed into short pieces (siRNA) by the endonuclease Dicer. The siRNA is loaded into the RNA-induced silencing complex (RISC) via the RISC loading complex (RLC), followed by cleavage and release of the passenger strand. The guide strand then associates with a homologous mRNA strand by conventional base paring, and the mRNA strand is cleaved by RISC and released for further degradation within the cytoplasm.

RNAi can be broadly defined as a class of processes that use short RNAs to regulate transcriptional and post-transcriptional gene regulation. Since the discovery of RNAi, numerous *in vivo* and *in vitro* studies have been performed to improve our understanding of the RNAi processes, and to identify the different components of the RNAi machinery of disease-causing organisms. From a combination of results, a widely conserved three step mechanistic model for RNAi has been derived (Figure 1) [13,14]. 

**Table 2:** Small RNA species and RNAi machinery described in vectors of disease and their associated transmitted pathogens. The human louse is not included as there is no current information on RNAi machinery in this organism.

Vector of disease/ Protozoan pariste	Class of small silencing RNAs	RNAi machinery				Reference
		Dicer (Drosha in miRNA pathways)	RISC complex	Transitive amplification	Systemic protein	
Vector						
Tsetse fly (Glossinidae)	Unexplored	ND	ND	ND	ND	[15,16,17]
Pathogen(s) transmitted						
*Trypanosoma brucei*	siRNA	TbDCL1 (cytoplasm, RNase IIIa)	TbAGO1, TbRIF5	Unknown	Unknown	[18,19,20,21,22]
		TbDLC2 (nucleus, RNase IIIb)	TbRIF4, PIWI-tryp			
Vector						
Triatomine or kissing bugs (Triatome/ Reduviidae)						
*Rhodnius prolixus*	Unexplored	ND	ND	ND	ND	[23]
*Triatomine brasiliensis*	Unexplored	ND	ND	ND	ND	[24]
Pathogen(s) transmitted:						
*Trypanosoma cruzi*	piRNA	Absent	PIWI-tryp	Absent	Absent	[25]
Vector						
Ixodid tick						
*I. scapularis*	miRNA	Drc-1	Ago-1 (PIWI and PAZ domain)	*Epn-1Cele	Rsd-3	[26]
	siRNA		Ago-2 (PAZ)	AP-50, Arf72, Clathrin hc, Rab7, CG3911, Cog3, IdICp		
Pathogen(s) transmitted:						
*Babesia, Borrelia, Anaplasma*	Unexplored	ND	ND	ND	ND	
Vector						
Mosquitoes						
*Aedes aegypti / A .albopticus*	miRNA	Drosha, Dicer-1 (Pasha, Loqs)	Ago-1 (x 2)		Lack SID-1, but shows systemic response	[27]
	siRNA	Dicer-2, R2D2	Ago-2 (VIG, TSN, Fmr-1)			
	piRNA	Absent	Ago-3, Ago-4 like (x 4), Ago-5 like (x 3)			
*Culex quinquefasciatus*	miRNA	Drosha, Dicer-1 (Pasha, Loqs)	Ago-1			
	siRNA	Dicer-2, R2D2	Ago-2 (x 2) ( TSN, Fmr-1)			
	piRNA	Absent	Ago-3, Ago-4 like (x 3), Ago-5 like (x 3)			
*Anopheles gambiae*	miRNA	Drosha, Dicer-1 (Pasha, Loqs)	Ago-1			
	siRNA	Dicer-2, R2D2	Ago-2 (Fmr-1)			
	piRNA	Absent	Ago-3, Ago-4 like, Ago-5 like			
Pathogen(s) transmitted:						
*Plasmodium*	Absent	Absent	Absent	Absent	Absent	[28,29]

The first step in allowing non-cell-autonomous RNAi is the uptake of the dsRNA, followed by an RNAi initiating step. It involves the binding of an RNA-specific nuclease to a dsRNA fragment and its cleavage into smaller double stranded small RNAs, usually of either the interfering (siRNA, from exogenous long dsRNA precursors) or miRNA (triggered by endogenous stem-loop pre-miRNA) type. The third step involves the loading of the small dsRNAs into a multi-nuclease complex. Finally, within this protein complex, the guide RNA strand associates with a homologous mRNA strand by conventional base paring, and the mRNA strand is cleaved and released for further degradation within the cytoplasm (Figure 1).The recent comparative genomics studies between the processes of RNAi in different invertebrate organisms and model organisms such as *D. melanogaster* and *C. elegans* have provided evidence that, even though much of the basic RNAi machinery is evolutionary conserved, several components differ, indicating that the RNAi pathway may vary substantially between different arthropod classes [26].

### 2.1. dsRNA Uptake

Initiation of the RNAi pathway takes place once dsRNA is present within the cell [32]. In the case of artificially introduced exogenous dsRNA, two mechanisms of dsRNA uptake have been described in invertebrates: transmembrane channel-mediated uptake through orthologs of the multi transmembrane protein SID-1 (systemic RNA interference deficient-1) of *C. elegans* and an endocytosis-mediated uptake mechanism [33]. 

SID-1 is a non-specific dsRNA transporter, engaged in the passive transport of dsRNA into cells and the spread of an RNAi response throughout an entire organism (systemic RNAi) [34,35]. This transporter protein is surface-expressed and displays diffusion-limited dsRNA transport that is energy independent. Recent evidence shows that SID-1 is instrumental in the import of silencing signals in *C. elegans* but not for export to neighboring tissues [36], and cells that are known to be refractory to gene silencing with exogenous dsRNA have been shown to be SID-1 deficient. Expression of SID-1 in these cells facilitated dsRNA uptake and subsequently an RNAi response [37]. While homologs of SID-1 have been reported in numerous organisms ranging from humans to various insects, they are noticeably absent from the genomes of others such as *D. melanogaster*, and this absence seems to correlate with a lack of a strong systemic RNAi response [34,38]. 

Endocytosis as an uptake mechanism originated from the observation that *D. melanogaster* cells do not express SID-1 but are still able to internalize exogenous dsRNA. Studies of *Drosophila* S2 culture cells resulted in the identification of several factors necessary for dsRNA uptake, of which many are also implicated in endocytosis [39]. Owing to the fact that the RNAi response was weakened by blocking endocytosis via pharmaceutical agents, the role of endocytosis in RNAi is evident [40]. Uptake experiments with FITC-labeled dsRNA conducted by Saleh *et al.* (2006) also showed that dsRNA molecules associate with vesicles for receptor-mediated endocytosis, with components such as vacuolar H^+ ^ATPases and scavenger proteins participating. It appears as if H^+ ^ATPase plays a crucial role in catalyzing an endocytic pathway for successful RNAi induction. Two scavenger receptors, Eater (which mediates phagocytosis of bacterial pathogens) and Sr-CI (a scavenger receptor class C, type I protein) facilitate uptake of over 90% of the dsRNA in *D. melanogaster* [39], demonstrating that receptor-mediated endocytosis a major avenue. The involvement of endocytosis in the uptake of dsRNA in *C. elegans* is also apparent with various “uptake-genes” being involved. RSD3 (RNAi spreading defect 3) seems to be involved in uptake via its ENTH-domains (Epsin N-terminal homology domain), which have been linked to several intracellular trafficking events [33].

### 2.2. Initiation of RNAi

The goal of the initiator step is to generate siRNAs from dsRNA. When exploiting RNAi as a reverse genetic tool, the process is artificially induced by the delivery of a dsRNA trigger. In non-mammalian systems long dsRNA (>200 bp) homologous to the target gene effectively triggers RNAi. In mammals, however, the introduction of dsRNA longer than 30 bp results in the activation of an anti-viral interferon response, which causes systemic non-specific inhibition of translation. Therefore in mammalian systems short synthetic siRNAs or DNA constructs, which express short hairpin RNAs (shRNAs), are typically used [41,42]. Since the specificity of RNAi depends on the sequence and structure of the siRNAs [30], it is of great importance to carefully design the dsRNA or shRNA-constructs in order to maximize silencing of the target gene and ensure minimal off-target effects.

The introduction of long dsRNAs into non-mammalian cells cause it to be processed into 21–25 nt double stranded RNAs (siRNAs), which have characteristic structures indicative of ribonuclease III (RNase III) cleavage [14,43,44]. Hannon and colleagues identified and demonstrated that the enzyme responsible for the initiation of RNAi in *Drosophila* is in fact an RNase III-like protein and owing to its biochemical function, named the enzyme Dicer [45,46]. Dicer acts as a monomer: the two ribonuclease III (RNase III) domains form a single processing center by assembling into a pseudo-dimer producing 2nt 3'-overhangs [47]. Dicer preferentially processes dsRNA from the ends of the substrates, with the Piwi/Argonaute/Zwille (PAZ) domain characterizing the 3'-overhangs [48,49,50,51]

More profound insight into the structural mechanism used by Dicer came from the crystal structure of a Dicer protein derived from the protozoan *Giardia intestinalis* [52]. This structure revealed that Dicer attains an elongated shape and by this means acts as a molecular ruler, measuring the distance between the terminal-binding PAZ domain and the active site, thereby giving rise to the 21–25 nt products [52,53,54]. Although the structure and mechanism of *G. intestinalis* Dicer has been studied in detail, much less is known about Dicer proteins from higher eukaryotes [55]. Furthermore, *G. intestinalis* Dicer is an atypical Dicer in that it is much smaller and simpler than that found in any other organism to date, containing only the two RNase III domains and a PAZ domain. For example, human Dicer (219 kDa) is nearly three times larger than that of *G. intestinalis* (82 kDa). The difference in molecular mass is accounted for by at least five additional protein domains found in most Dicer proteins [55]. These include a TAR RNA-binding protein (TRBP) [56], an amino terminal DExD helicase-like domain [57], a putative dsRNA-binding domain (dsRBD) named DUF283 [58], an Argonaute-binding domain [59] and a C-terminal dsRBD [60]. These additional domains participate in dsRNA processing, regulate dicing activity and serve as molecular scaffolds for consolidating protein factors involved in the initiation of RNAi [55].

### 2.3. RNA-Induced Silencing Complex (RISC) Assembly

Once a dsRNA trigger has been processed into siRNAs, the guide strand is identified and loaded into an effector ribonucleoprotein complex, the RNA-induced silencing complex (RISC). Since siRNAs cannot catalyze any reaction by themselves, RISC assembly is a key process for these small RNAs to exert their function [61]. To date, a complete understanding of the exact mechanism underlying this process remains elusive. Based on several *in vitro* studies of RISC assembly using *D. melanogaster* extracts and siRNAs as substrates, together with new structural studies of Argonaute (Ago), a revised 2-step model of the main features of this process has been proposed [62,63]. The first step, known as RISC-loading, involves the insertion of the siRNA duplexes into the Ago-protein. The second step entails the dissociation or unwinding of the passenger strand from RISC [61].

It is postulated that Dicer and a dsRNA binding partner (e.g. in *D. melanogaster* these are Dicer-2 and R2D2) are bound to the double stranded siRNAs, to direct them to the RISC complex [64]. Current findings suggest that Dicer-2 and R2D2 form a heterodimer that binds the siRNA, with Dicer binding the thermodynamically less stable end of the siRNA and the dsRBD domain binding the more stable end [61,65]. These thermodynamic and binding asymmetries appear to determine which strand is finally incorporated into RISC. The guide strand is always the strand whose 5’ end is less tightly paired to its complement, implying the strand with the least thermodynamically sTable 5’ end [66,67]. Once the Dicer/dsRBD/siRNA ternary complex is formed it assembles with Ago-protein to form a RISC loading complex (RLC), which is an intermediate from which Dicer/dsRBD is gradually displaced by the Ago-protein to form pre-RISC [61,64,65,68,69]. Once bound to the siRNA duplex, Ago cleaves the passenger strand, triggering its dissociation [70]. After discharge of the passenger strand, possibly in an ATP-dependent step, the guide strand is in close association with Ago, forming a fully mature RISC, also referred to as siRISC [61,64,65,71].

### 2.4. Slicing or Silencing Steps

In *D. melanogaster*, several RISC components have been identified, but only a few have been characterized at the functional level [72]. From current findings it is evident that RISC consists of several proteins and RNA molecules that act as key actors of RNAi promoting mRNA degradation, repression of translation and remodeling of chromatin structure [73]. The central catalytic component of RISC, Argonaute 2, was the first to be identified [74]. Argonaute proteins are characterized by two conserved domains: the PAZ (also present in Dicer) and PIWI domains [75]. Based on a higher degree of homology to either *Arabidopsis* AGO-1 or *Drosophila* PIWI, this family of proteins is subdivided into two highly conserved subclasses [76]. Recently an additional subclass, *C. elegans-*specific group 3 Argonautes, has been identified [77]. Other components which have been identified as part of the RISC-complex include the Vasa intronic gene (VIG)-RNA binding protein, helicase proteins, Tudor- staphylococcal nuclease (Tudor-SN) and Fragile X -protein (dFXR) homologs [78,79]. To date most of these proteins remain functionally uncharacterized, however. Due to the lack of structural data, the exact catalytic mechanism of target mRNA cleavage remains unclear. 

In the RISC complex the 3’ end of the siRNA guide strand is bound by the PAZ domain, while the PIWI domain contacts the 5’ phosphorylated end [49,50]. Structural studies of prokaryotic Ago-like protein in complex with siRNA-mimics demonstrated that the sugar-phosphate backbone of the 5’ end of the guide strand (nucleotide 2 to 8) contacts the PIWI domain in such a manner that bases 2 to 8 are presented on the surface [80,81]. This region is known as the ‘seed’ region [14]. Corresponding mRNA targets are initially bound by the seed region of the siRNA and then pairing is extended to the 3’ end. A single cleavage of the target mRNA occurs across from nucleotide 10 and 11 (with respect to the 5’ end) of the guide stand. The catalytic engine is an RNaseH fold present in the PIWI domain [75,81,82]. Although target cleavage by Argonaute does not require ATP *per se*, release of the cleaved target (RISC recycling) is stimulated by ATP [83,84]. From *in vivo* studies it is evident that, once released from RISC, the 3’ mRNA fragment is degraded in the cytoplasm by exonuclease Xrn1, while the 5’ fragment is degraded by a complex of exonucleases, the so-called exosome [85]. In the RNAi pathway, siRISC operates as a multiple turn-over enzyme. Once the mRNA target is cleaved, siRISC dissociates from the cleaved mRNA to repeat another cleavage cycle [30].

In plant and nematode species it has been described that the RNAi signal is amplified by an RNA-dependent RNA polymerase (RdRP), which results in a systemic RNAi effect that can spread from the targeted/treated tissue to other tissues [86]. To date, this RdRP-dependent RNAi amplification seems absent in higher eukaryotes, and even from *D. melanogaster* and mosquitoes. However, based on several RNAi studies in ticks it is evident that this phenomenon does occur in ticks, since application of dsRNA (via body cavity injection, feeding or soaking) results in global and persistent gene silencing in both treated ticks and their progeny [87,88,89,90]. Recent preliminary studies have indicated that this systemic RNAi effect in ticks is most likely attributed to a tick RdRP homolog [26]. 

**Figure 2:**
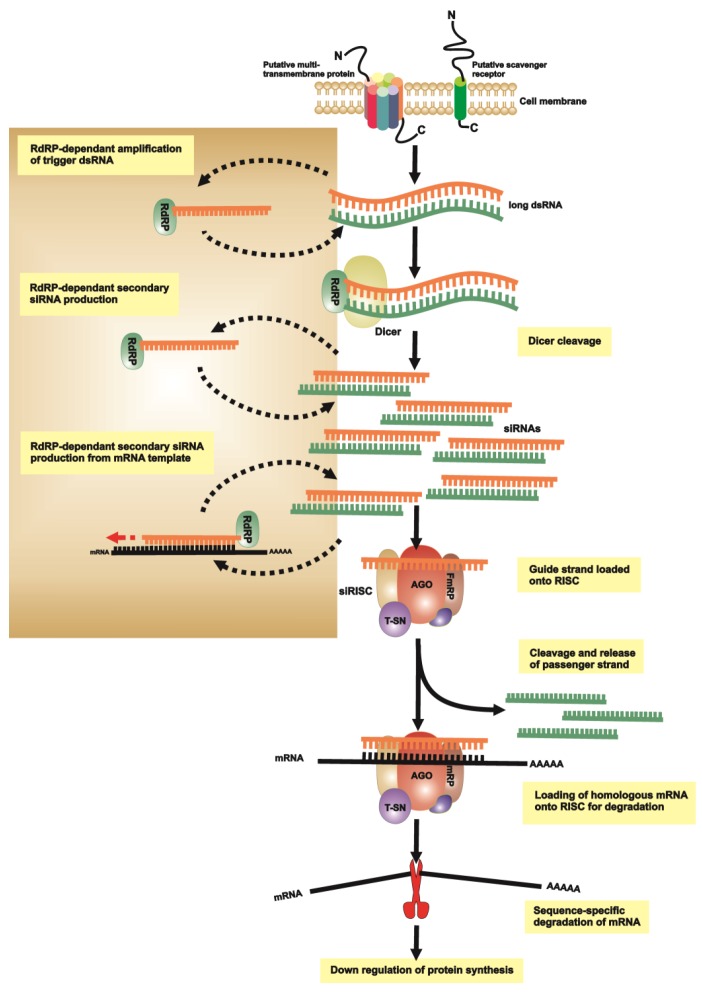
A schematic representation of a putative RNAi pathway for invertebrate pathogens and vectors. The proposed model might either use a multi trans-membrane protein (similar to SID-1) for dsRNA uptake or an endocytosis-mediated process which may include scavenger receptors. Once the dsRNA is in the cytoplasma it is processed into siRNAs ~ 21–23 nt in length, by a Dicer homologue. The siRNA are then presented to RISC which incorporates the siRNA, targets and degrades any homologous mRNA. RISC includes Ago, TudorSN and FmRp homologues. The proposed activity of RdRP is indicated as amplifying either trigger dsRNA, cleaved siRNA or using primary siRNA to prime synthesis of secondary siRNAs utilizing native mRNA as template. This causes 5’ amplification and spreading of the initial RNAi signal, and is known as transitive RNAi.

In order for RNAi to spread from one cell to another a transport system is required. In *C. elegans* several proteins, necessary for systemic RNAi have been identified. These include a multi transmembrane protein SID-1 (systemic RNA interference deficient-1(sid-1)) thought to act as a channel for dsRNA uptake [34], as well as RNAi spreading defective proteins (RSD2, RSD3, RSD6) [91]. To date only RSD3 and Endocytic protein (Epn-1) have been identified in ticks, leaving the mechanism of cell to cell dsRNA transport in ticks unresolved. Although more components of the tick RNAi machinery have been identified, the exact mechanism remains to be determined [26]. The current model for most invertebrate parasites RNAi is based on integrated knowledge of the RNAi process of model/other organisms including, *D. melanogaster, C. elegans* and mosquitoes (Figure 2). 

### 2.5. Transitive Amplification of the Initial dsRNA Signal

In various plant and nematode species it has been found that the silencing signal is amplified by a process reliant on a RNA-dependent RNA polymerase (RdRP), which in effect permits a systemic RNAi response from the site of origin to eventually most or all other tissues [26,86]. A two-step mechanism has been described for RdRP amplification in *C. elegans* [92]. Once the primary siRNA has been generated by Dicer, RdRP uses the guide siRNA strand as primer and native mRNA as template to synthesize abundant secondary siRNAs and increasing the effectiveness of RNAi (Figure 2). As RNA synthesis occurs in a 5'–3' direction, this amplification leads to the 5' spreading of the initial RNA interference signal. This phenomenon is known as transitive RNAi [92]. 

To date RdRP-dependent RNAi amplification seems absent in *D. melanogaster*, many other insects and higher eukaryotes. Interestingly, a putative homolog of the RdRP EGO-1 protein from *C. elegans* has also been identified in the tick species *I. scapularis*. This protein is associated with the amplification of trigger RNA by transcribing additional dsRNA molecules using target mRNA as a template, thereby yielding secondary siRNAs and increasing the effectiveness of RNAi [40,93]. RdRPs appear to be absent in other metazoans except for several nematodes and a lancelet (*Branchiostoma floridae*) [94]. The tick RNAi pathway may therefore differ from that of other arthropods, but more research is required to clarify the exact RNAi mechanism.

### 2.6. Systemic RNAi

In order for RNAi to spread from one cell to another a transport system is required. The phenomenon of the progression of an RNAi response from the site of origin to neighboring cells and eventually to all, or most, tissues of the organism was first described in plants and *C. elegans* . The systemic response may even persist through multiple developmental stages, including being transferred through the germline to the progeny. It has been proposed that this response has evolved as a strategy to increase the silencing efficiency [95] and it is known to form part of the immune system in plants. For the functioning of a robust systemic response there are three essential steps: firstly the uptake of an extracellular dsRNA signal into a cell, secondly the amplification and distribution of the said signal to other cells, followed by a successful degradation or blocking of a homologous mRNA transcript [38].

Although the exact mechanisms pertaining to a systemic RNAi response remain unclear, many benefits associated with its presence makes it a highly desirable feature in organisms investigated using RNAi. Firstly, the amplification of the introduced RNAi signal means that lesser/smaller amounts of dsRNA can evoke the needed response throughout the entire organism. Secondly, the fact that silencing is spread and effected throughout all tissues makes target gene selection of the whole genome of the studied organism possible. This is especially relevant in those experiments where the mode of administering dsRNA cannot deliver it to all tissues, for example through oral delivery or direct injection. Another application made possible is the study of genes in life stages such as eggs that may not be amenable to other methods of dsRNA delivery. By delivering the relevant dsRNA to a preceding life stage, in this case the adult female, systemic RNAi will propagate the necessary signal throughout the adult as well as within any eggs produced by that female [88]. This application is known as parental, trans-stadial or transovarial RNA interference. Exploiting systemic RNA interference during RNAi studies is also a relatively simple procedure. For these reasons there is a definite advantage and incentive to elucidate the complete mechanism of systemic RNAi so that it could be induced experimentally in organisms that do not show a systemic RNAi response themselves.

Although more pieces of the RNAi machinery of different disease vectors and pathogens have been identified, the exact mechanism of each remains unknown. Current RNAi models for most invertebrate parasites is based on integrated knowledge of the RNAi process of model/other organisms including *D. melanogaster, C. elegans* (Figure 2). 

## 3. RNAi of Lice, Ticks and Tick-Borne Pathogens

### 3.1. RNAi Pathways and Methodologies

Apart from model organisms such as *Drosophila*, ticks have provided us with a significant understanding of RNAi in arthropod vectors of disease and hence will be discussed in a bit more detail in this section. Ticks are obligate hematophagous parasites and are important vectors of a wide variety of pathogens including viruses, protozoa and bacteria which affect human and animal health worldwide [96]. Elucidating tick-host-pathogen interactions is of interest for the development of novel intervention strategies such as anti-tick and pathogen-transmission blocking vaccines to limit the damage caused by ticks and prevent the spread of pathogens. The increasing availability of genomic data from tick-borne pathogens and ticks such as *Ixodes scapularis* and *Rhipicephalus (Boophilus) microplus* has introduced a new era in tick research and greatly advanced studies addressing tick biology and host-vector-pathogen interactions [97,98,99]. The possibility to silence the expression of genes in ticks using RNA interference (RNAi) has proven to be pivotal in numerous studies which focus on deciphering tick gene function. Since the first report of an RNAi experiment in *Amblyomma americanum* ticks in 2002 [100], this method has been applied successfully in other hard ticks belonging to the *Amblyomma*, *Dermacentor*, *Haemaphysalis*, *Ixodes*, and *Rhipicephalus* genera and in *Ornithodoros* soft ticks [101,102,103].

Mites belong to the same subclass as ticks (Acari) and may also act as vectors of disease, albeit to a lesser extent. The most important mite-borne disease of humans is scrub typhus (*Orientia tsutsugamushi*), which is primarily transmitted by several members of the genus *Leptotrombidium*. Parasitic mites living on or burrowing in the skin may also cause dermatitis in humans and animals. The full genome of the two-spotted spider mite *Tetranychus urticae*, a plant-feeding mite, was recently published and is the first chelicerate genome to be completely sequenced [104]. Successful gene silencing by RNAi has also been described for this mite and the honey bee mite *Varroa destructor* [105,106].

Two mechanisms of dsRNA uptake have been described in arthropods: a transmembrane channel-mediated uptake through orthologs of the SID-1 protein of *C. elegans* and an endocytosis-mediated uptake [33]. As SID-1 is not present in tick and mite genome sequence databases, an endocytosis-mediated uptake is hypothesized in Acari. In the endocytosis-mediated uptake of dsRNA in *Drosophila*, scavenger receptors are thought to mediate dsRNA uptake [39,40]. Evidence for an endocytosis mediated mechanism for dsRNA uptake with an essential role for a scavenger receptor in ticks was recently provided by an ‘RNAi of RNAi’ approach. When the expression of *HlSRB*, the class B scavenger receptor CD36 from *Haemaphysalis longicornis,* was silenced 96h before dsRNA coding for a second target gene (*HlVg-1* or *HlVgR,* encoding vitellogenin and vitellogenin receptor) was introduced, subsequent knockdown of the second gene was inhibited. A double knockdown of target genes could however be observed when *HlSRB* was silenced 96h after the knockdown of *HlVg-1* or *HlVgR* [93]. This suggests that receptor-mediated endocytosis is also relevant for systemic RNAi in ticks, with an essential role for scavenger receptor class B. Orthologs of the class B scavenger receptor CD36 within the superorder Parasitiformes have been annotated for *I. scapularis* (GenBank Accession number XP002409323) and the western predatory mite *Metaseiulus occidentalis* (XP003743396). Other key components of intracellular RNAi were recently identified from tick and mite genomic sequence databases by homology searches [26,104]. These include putative Dicers responsible for the cleavage of dsRNA to siRNAs, Argonaute proteins which function as the catalytic component of RISC and others (Table 2). 

RNAi is usually accomplished by treatment of ticks, tick tissues, tick cells or mites with dsRNA, but the successful use of siRNA to silence gene expression in these specimens has also been reported [106,107,108,109]. Most laboratories transcribe dsRNA *in vitro* from a PCR template which has been amplified using primers containing a T7 promoter sequence. The relative simplicity of synthesizing dsRNA and its high efficacy have made it the most commonly used mediator in tick RNAi studies. A drawback of the use of long dsRNA is that it increases the chance of causing off-target effects (OTE), leading to false discovery. OTE are caused by species-specific recognition of transcripts other than the intended target. This can be addressed in the experimental design by preventing the use of dsRNA sequences of which nucleotide stretches may recognize non-target transcripts. Various *in silico* prediction algorithms to analyze possible OTE have been described [110,111] but their application in tick research is frustrated by the absence of full tick genome data. The use of siRNA, small dsRNA (100–200 bp) or of two non-overlapping dsRNAs per gene to assess whether both induce the same phenotype, may limit the occurrence or facilitate the detection of OTE [110,112]. However, these strategies have thus far found only limited adaptation in tick research using RNAi.

The injection of dsRNA into the hemacoel via the ventral exoskeleton surface using fine-gauge or pulled capillary needles is the most commonly used delivery method for dsRNA in ticks. After a recovery period to monitor for tick mortality resulting from the injection, ticks are fed on experimental animals or an artificial feeding system [100,113]. Parameters such as tick mortality, tick weight after feeding and the amount of eggs oviposited can subsequently be recorded to evaluate the effect of gene silencing on general tick function. Biological material such as hemolymph or saliva and tissues can furthermore be collected for further analysis in functional assays and histological examination [114,115,116,117]. By injecting dsRNA into the hemocoel of freshly engorged female ticks through the spiracular plate, gene silencing can be established in the eggs and larvae hatching from these eggs [88,118,119]. A similar method can be used to silence genes in the eggs of mites [106]. The spread of dsRNA to different tissues following injection in the hemacoel demonstrates that RNAi is systemic in the Acari lineage. 

Infection of the *I. scapularis* cell line ISE6 with the a recombinant Semliki Forest Virus replicon expressing heterologous Nairovirus sequences induced siRNAs which inhibited the replication of Hazara virus, a Nairovirus, by RNAi in this cell line [120]. Besides this viral delivery, the incubation with dsRNA or siRNA is also effective in establishing gene silencing by RNAi in tick cells. Optimum conditions were however shown to vary for different cell lines. DsRNA was more efficient for gene silencing by RNAi in tick cells than siRNA and use of the latter requires a transfection reagent [121]. RNAi can also be used to study the effects of gene silencing on tissue function *ex vivo* following the immersion of dissected tissues in a dsRNA solution [113,122,123]. Overnight soaking at 4 °C in a 0.9% saline solution containing dsRNA at a concentration of 2,5 µg/µL has been applied successfully to establish gene silencing in whole *Varroa destructor* mites. The addition of NaCl was apparently necessary as soaking in plain water containing dsRNA was not effective [105]. The soaking of whole ticks, which would reduce the laboriousness and mortality associated with injections and simplify dsRNA delivery especially in juvenile tick stages, has also been reported for *Haemaphysalis longicornis* nymphs but requires further optimization [124]. Soaking of eggs and nymphs in combination with electroporation was successfully used for dsRNA delivery in *I. scapularis* ticks [125]. 

Further evidence for the occurrence of systemic RNAi in ticks was provided by Soares *et al.*, who delivered dsRNA to *I. scapularis* nymphs using capillary feeding of a dsRNA solution. Ingested dsRNA targeting an anticomplement gene (*isac*) in the salivary gland spread via the gut to the target organ, where it resulted in a knockdown of *isac* expression [89]. 

### 3.2. RNAi to Understand Tick Vector Biology

RNAi has also helped to unravel the molecular complexity of tick physiology and biology. Hard ticks for example concentrate their blood meal during feeding by secreting excess water imbibed with the blood back into the host with saliva. A water channel or aquaporin (AQP) was found to be expressed in the gut and salivary glands, organs which are associated with a high water flux. Gene silencing of AQP in *I. ricinus* females by RNAi disrupted the water passage from gut to salivary glands, resulting in an accumulation of blood in the gut which could not be concentrated. The gut subsequently became too full to accommodate more blood and the total blood volume ingested was significantly reduced. The hemolymph osmolarity consequently increased and fluid secretion by isolated and ligated salivary glands was abolished [113]. Other examples of biological and physiological processes which have been studied in ticks include blood feeding [89,115,124,126,127,128,129,130,131], blood digestion [132,133], iron metabolism [134], reproduction [135,136] and innate immunity [137].

Another interesting application of RNAi in ticks is its use as a tool to screen for tick protective antigens which may be suitable anti-tick vaccine candidates [138,139]. In these screens, dsRNA is transcribed from individual clones of a cDNA library and screened in increasingly smaller pools for their effect on tick feeding and survival. This may result in the identification of proteins that are critical for the tick which can subsequently be evaluated as potential vaccine antigens. By using this approach, researchers could identify subolesin, a protein which was shown to have efficacy as a recombinant antigen in vaccination trials against various tick species, the poultry red mite *Dermanyssus gallinae,* mosquitoes and sand flies [140]. 

Silencing of subolesin produces ticks with diminished reproductive performance which prevents successful mating and the production of viable offspring. The release of subolesin-silenced ticks, alone or in combination with subolesin vaccination, was proposed as an alternative tick control strategy [119,141]. Although interesting, many questions concerning the feasibility and consequences of this approach, such as the reduced fitness of modified ticks and the ecological impact of their release, remain to be answered before this or similar methods can be put into practice. 

Injection of adult female mites or engorged female ticks with dsRNA results in gene silencing in their offspring, a phenomenon which is also known as ‘parental’ or ‘transovarial’ gene silencing [88,106,118]. Parental RNAi may be used to study the function of genes in embryogenesis and will facilitate studies in evolutionary developmental biology, an area of tick and mite research which lags behind that of many other invertebrates. 

### 3.3. RNAi to Understand Tick-Pathogen Interactions

To study tick-pathogen interactions, a typical experimental approach starts with the identification of tick genes which are differentially expressed when ticks are infected with a pathogen. Techniques such as suppression subtractive hybridization (SSH) [142], two-dimensional (2-D) gel electrophoresis [143], 2-D fluorescence difference gel electrophoresis (DIGE) [127], RT-PCR [144] and microarrays [145] have been used for this purpose. The differential expression is usually confirmed by (semi-)quantitative RT-PCR. The expression of genes which are found to be up-regulated following infection can subsequently be silenced by RNAi to study the effect of gene knockdown on the course of the infection. This approach was for instance followed to identify tick salivary gland proteins up-regulated in *I. scapularis* ticks in response to infection with *Borrelia burgdorferi*, the causal agent of Lyme disease of which *I. scapularis* is the predominant vector in North America. DIGE coupled with mass spectrometric analysis using extracts from *B. burgdorferi*-infected and uninfected salivary glands led to the identification of a histamine release factor (*tHRF*) which was strongly up-regulated in the salivary glands of *B. burgdorferi*-infected ticks during engorgement. Gene silencing of tHRF in *B. burgdorferi*-infected nymphs impaired successful feeding and resulted in reduced spirochete levels in ticks and the mice they had fed upon. It is suggested that the decreased feeding ability of tHRF-deficient ticks may have disrupted the *B. burgdorferi* replication and dissemination inside the ticks, reducing the efficiency of spirochete transmission [127]. The laboratory of Erol Fikirg, also identified 14 different salivary gland proteins (salp) which are secreted into the host during feeding, by probing an *I. scapularis* salivary gland cDNA expression library with serum from tick-immune rabbits [146]. The expression of two of these genes, *salp15* and *salp16*, was found to be selectively increased in *I. scapularis* ticks infected with *B. burgdorferi* or *A. phagocytophilum,* respectively [144,147]. Gene silencing of *salp15* and *salp16* did not impair tick feeding as tHRF did, but did interfere with the pathogen life cycle. While the *B. burgdorferi* levels in the salivary glands of control and *salp15*-deficient ticks were similar, the spirochete burden was significantly lower in the mice on which *salp15*-deficient ticks had fed. This demonstrated that Salp15 facilitates tick-borne *B. burgdorferi* infection in the host [147]. Silencing the gene expression of *salp16* inhibited the acquisition of *A. phagocytophilum* by the vector tick from infected mice. Pathogen levels in the gut of control and salp16-deficient ticks which had fed on infected mice were similar, but the *A. phagocytophilum* levels in salivary glands were significantly lower in *salp16*-silenced ticks, suggesting that *A. phagocytophilum* requires *salp16* to infect the salivary glands [144]. Other functional analysis studies based on RNAi which focused on the tick immune responses have also identified genes which may be involved in the vectorial capacity of ticks for a variety of pathogens [114,142,148,149].These examples demonstrate the intimate relationships between vector-borne pathogens and their arthropod hosts on which RNAi shed light. 

### 3.4. RNAi in Phthiraptera (Lice)

Lice are obligate ectoparasites of mammals and birds which may feed on blood (bloodsucking lice of the suborder Anoplura) or on skin debris, hairs and feathers (chewing lice). Three Anoplura species are ectoparasites of humans: the head louse (*Pediculus humanus capitis*), the human body louse (*Pediculus h. humanus*) and the crab louse (*Phthirus pubis*). Of these three, only the body louse is a vector of pathogens, including the causal agents of epidemic typhus (*Rickettsia prowazekii*), trench fever (*Bartonella quintata*) and louseborne relapsing fever (*Borrelia recurrentis*) [150]. The genome of the body louse was recently sequenced and is with 108 Mb the smallest insect genome known to date [151]. A gene-specific reduction in transcript levels following the injection of dsRNA in female lice was recently reported [152] and key components of the RNAi pathway have also been identified in the human body louse genome [150]. Their small genome size, the presence of a functional RNAi pathway and differences in their ability to transmit pathogens make the head and body louse interesting models for comparative functional genomic studies to unravel the molecular mechanisms which determine vector competence [150].

## 4. RNAi of Mosquitoes and Their Associated Pathogens

Only a few mosquito species are vectors of pathogens that cause serious disease. *Anopheles gambiae* is the primary vector for the malaria parasite *Plasmodium falciparum*. *Aedes* species are responsible for potentially fatal encephalitis and hemorrhagic fevers caused by Dengue and Yellow fever viruses and certain *Culex* species transmit West Nile virus and nematodes that cause filariasis in humans (Table 1). The capacity of mosquitoes to serve as vectors revolves around a number of essential behavioral and biological factors, such as their preference for blood-feeding, their high susceptibility to parasite infection and their longevity [153]. Some of the most important vector species require multiple blood meals during each egg-laying cycle, thereby increasing the risk for pathogen transmission [154]. 

### 4.1. dsRNA Introduction into Mosquitoes

RNAi-based silencing of gene expression has been achieved in mosquitoes using a number of different dsRNA delivery methods. In the majority of studies to date, *in vitro* synthesized dsRNA have been injected directly into the mosquito hemolymph. Those that survive the micro-injection procedure are then fed or exposed to the relevant physiological condition and/or stress factors and evaluated for the phenotypic effect caused by knockdown of the target gene. In mosquitoes, these often relate to effects on certain aspects of fecundity such as oviposition and egg development, or physiological evidence of cell damage, insect mortality or behavioral changes (reviewed in [155,156]. The micro-injection procedure, however, is not optimal or efficient particularly in light of the mosquito’s small size. One alternative is to use oral introduction, as demonstrated in a study where chitin synthase-targeted dsRNA was fed to *An. gambiae* larvae, resulting in significant mortality [157]. Another is by topical application of dsRNA, demonstrated in *Ae. aegypti* adults [158]. In a first, Coy *et al.* recently demonstrated that knockdown can be achieved in adult mosquitoes by the oral delivery of dsRNA via a liquid sucrose meal. The dsRNA was ingested by adult females and caused a reduction in the level of transcription of the vacuolar ATPase gene target [159]. A recombinant densovirus-mediated RNA interference system has also recently been developed for both *in vitro* and *in vivo* use [160]. The efficiency of RNAi has been shown to differ between tissue types, with midgut tissues and hemocytes being amenable to effective knockdown whereas inhibition of genes in the salivary glands required much higher dosages of dsRNA. Nonetheless, the larger quantities appeared to not compromise the silencing specificity [161]. Details of the uptake mechanism of exogenously added dsRNA and its subsequent processing has not been resolved yet, but processing is thought relate to the intrinsic virus-mediated RNAi response of mosquitoes (summarized below and reviewed in [33,162].

### 4.2. RNAi to Understand Mosquito Vector Biology

Functional studies using RNAi have provided a significant amount of insight into vector biology, such as integrated pathways that respond to hormonal and nutritional signals affecting feeding, vitellogenesis and egg production [153,163,164,165,166,167]; factors involved in lipid biosynthesis [168], vesicle transport between the ER and Golgi [169], steroid metabolism [166,170,171]. A large portion of literature also addresses olfaction in mosquitoes that influences aspects of their behavior, such as host seeking, feeding, mate selection and location of appropriate breeding sites [172,173]. Erdelyan and colleagues (2012) identified two CO_2_ receptor genes, AaGr1 or AaGr3 that upon knockdown resulted in a loss of CO_2_ sensitivity in *Ae. aegypti* and *Culex pipiens quinquefasciatus* mosquitoes, impacting host detection. Interruption of the olfactory system could be used to design compounds that disrupt the mosquito's ability to find a host, thereby limiting the potential for pathogen transmission [174]. 

### 4.3. RNAi as a Tool for Studying Mosquito Vector-Pathogen Interactions

Initial studies supporting RNAi-based gene silencing as a suitable tool for understanding the vector-pathogen interface in mosquitoes, include Dengue virus (DENV) replication inhibition by transfection of an *Aedes albopictus* cell line using DEN1- and DEN2-specific dsRNAs [175,176] and knockdown of the antimicrobial gene defensin by *in vivo* injection of gene-targeted dsRNA into the thorax of adult *Anopheles gambiae* [177]. A different approach [178], where dsRNA was expressed *in situ* from a transgene stably integrated into the mosquito genome and designed to transcribe an intron-spliced RNAi hairpin loop from an upstream promoter, have also become of great use. A myriad of studies have been reported, with most using direct delivery of dsRNA rather than the more demanding approach of transgenesis. 

With regard to viral transmission by mosquitoes, the long-standing view that mosquito innate immunity likely plays a role in restricting virus replication has been addressed in a number of studies. Based on the knowledge of immunity in *D. melanogaster*, orthologs of genes encoding Dcr2, R2D2 and Ago2, components of the intrinsic arthropod RNAi pathway, were silenced in a number of independent studies. Suppression of the mosquito RNAi defense mechanism resulted in an increase of alphavirus- and DENV replication in *Aedes aegypti* cultures and mosquitoes [27,179] and O’nyong-nyong replication in *Anopheles gambiae* [180]. These and other studies have provided clear evidence that RNAi is the major defense mechanism against arbovirus infections [181]. Some evidence has also been presented that the RNAi response may be systemic. In cultured *Aedes albopictus* cells, direct cell-to-cell spread of an RNAi signal inhibited the replication of incoming Semliki Forest viruses [182]. A similar spreading signal has been observed *in vivo* in *D. melanogaster* but the mechanism remains to be characterized. Studies have also provided evidence for the production of virus-derived piRNAs following arbovirus infection in mosquito cells, suggesting that this pathway may be another facet of antiviral defense [183]. It has also been possible to generate DENV2-resistant *Ae. aegypti* mosquitoes by constitutively triggering the RNA interference pathway via long DENV-specific dsRNAs transcribed from an integrated transgene. This transgenic approach provided heritable resistance to virus replication and transmission across 17 generation [184] before expression of the effector gene was then lost for unknown reasons. This encouraging result illustrates the potential of anti-pathogen suppression using RNAi, as explained in more detail in section 7.

Microarray-based transcriptional profiling in combination with RNAi is providing new information on immune signals that respond to virus infection. In one such a large-scale study, DENV infection of *Ae. aegypti* mosquitoes resulted in the identification of more than 70 genes putatively associated with immune signaling [185]. Knockdown of candidates has shown that both the Toll pathway [185] and JAK-STAT pathway [186], conventionally associated with defense against bacteria and fungi, also contribute to *Ae. aegypti* virus responses [187,188]. A recent study by Sim and Dimopolous provided evidence that Arboviruses are capable of actively suppressing the immune pathways in cells they infect, rather than simply failing to trigger host immunity [189]. When the transcriptome of *Ae. aegypti* cells infected with DENV was analyzed using microarrays, the Toll pathway, as in mosquitoes, was activated. In addition, numerous immune signaling molecules and anti-microbial peptides (AMPs) were down-regulated. In a similar study, Colpitts *et al.* also demonstrated down-regulation of four cecropins and a defensin in *Ae. aegypti* mosquitoes infected with respectively DENV, WNV and Yellow fever virus [190]. The molecular details of the findings in these studies have yet to be validated by functional RNAi studies. 

Finally, mammalian cells infected with DENV undergo apoptosis due to a shutting down of protein synthesis in host cells. In mosquito cells, however, apoptosis is mostly inhibited, likely to allow for the spreading of viral progeny to neighboring cells while evading host inflammatory responses [191]. Chen *et al.* provided evidence that mosquito cells also have strategies for protecting themselves against stresses induced by virus infection, such as GST that is involved in antioxidant defenses and an inhibitor of apoptosis, IAP. When GST was silenced in DENV-infected C6/36 mosquito cells, there was a higher rate of apoptosis [192]. Furthermore, silencing of IAP rendered mosquito cells highly sensitive to DENV-induced oxidative stress [193]. 

There is considerable interest in understanding the molecular and cellular interactions between mosquito vectors and protozoan parasites, with many studies focusing on the interaction between *An. gambiae* and *Plasmodium* species. In the past decade, RNAi approaches have been applied in numerous studies to unravel the genetic basis of the mosquito’s susceptibility and the immune responses elicited by the parasite. A multitude of mosquito genes that respond to *Plasmodium* infection have been identified in recent years as reviewed in a number of papers [194,195,196]. These include genes that may either negatively influence *Plasmodium* development, *i.e.* antagonists, or have a beneficial effect on parasite development, *i.e.*, agonists. Some of the first *An. gambiae* genes identified and verified by RNAi to encode anti-parasitic factors were TEP1 [197] and LRIM1 [198]. These are both pattern recognition receptors (PPRs) of the innate immune system, with TEP1 being a complement-like protein and LRIM1 a C-type lectin. RNAi-mediated silencing of TEP1 prevented melanization of parasites, a mosquito response to lower parasite load, while silencing of LRIM1 resulted in a substantial increase in oocyte numbers. APL1, a leucine-rich repeat protein was also verified as an antagonist [199]. Knockdown studies have since indicated that LRIM1 and APL1C occur as a heterodimer in the mosquito hemolymph that interacts with TEP1 [200,201]. It therefore seems as if the LRIM1/ApL1C/TEP1 complex’s function is to target *Plasmodium* parasites for destruction. 

Proteins expressed in the mosquito salivary gland that are involved in parasite invasion have also been characterized. A study of Gosh and colleagues demonstrated that interaction between the salivary-gland-specific surface protein saglin and the parasite surface protein TRAP (Thrombospondin-related anonymous protein) is essential for spoorozoite invasion. *In vivo* RNAi of saglin results in a strong inhibition of salivary gland invasion by parasite [202]. Many antagonists have since been discovered and their function analyzed using RNAi-based techniques. Most notably, these include the hypervariable PPR AgDscam [203], members of the fibrinogen-related protein family (FREP) [204] and the C-type lectin CLSP2 [205].

Gene silencing approaches have further shown that *An. gambiae* expresses proteins that protect *Plasmodium* from its immune defenses, or aid in their transport through membrane barriers. The C-type lectins CLT4 and CTLM2A serve as examples of immune system agonists. Gene knockdown / RNAi-mediated silencing of these genes results in massive melanization of ookinetes [198]. Some examples of mosquito proteins required for *Plasmodium* midgut and salivary gland invasion include AgESP, epithelial serine protease and upstream regulator of cytoskeleton remodeling [206], PRS1 (*Plasmodium* responsive salivary 1), a protein up-regulated in infected salivary glands [207], the antibacterial protein lysozyme c-1 [208], and hemolymph lipid transporter, ApoII/I [209]. RNAi-mediated silencing of these genes in some way caused a phenotype that validated their involvement in aiding *Plasmodium* invasion, reproduction and/or transmission. Proteins with properties like these are obvious candidates that merit further investigation for possible use in future anti-malaria strategies.

## 5. RNAi in *Triatominae* and *Trypanosoma cruzi*.

Only three examples of RNAi in Triatominae can be found in the literature to date. In 2006, salivary nitrophorin 2 (NP2) was silenced and both anticoagulant and apyrase activity in saliva were affected, confirming the role of NP2 in blood coagulation [23]. Insight into embryonic development of *Rhodnius prolixus* was provided by RNAi of the gap-gene *Rp-gt,* confirming its role in proper head and abdomen formation and that the function of *gt* in hemipterans is more similar to that in dipterans than previously anticipated. This will be invaluable for understanding the evolution of patterning in insect embryos [210].In the closely related *Triatoma brasiliensis*, an intestinal thrombin inhibitor (brasiliensin) was silenced in nymphs [24,211]. Knockdown reduced both the thrombin inhibitory activity and the plasma clotting time in midgut tissue, confirming brasiliensin as a potent anti-haemostatic targeting thrombin. In addition, silencing decreased bloodmeal uptake, suggesting that anticoagulant activity in the vector midgut influences the amount of blood taken from the host. With the commencement of mass sequencing of the salivary glands of triatomines, many novel genes of unknown function have been identified and need to be evaluated as possible targets for control. With phenotypical analysis, after the introduction of dsRNA by injection or ingestion, it was evident that although the relevant machinery has not been elucidated to date, an RNAi pathway is present in *R. prolixus* allowing functional genomic studies.

*Trypanosoma cruzi*, the causative agent of South American trypanosomiasis / Chagas Disease, is transmitted by *R. prolixus* and is regarded as a neglected tropical disease affecting millions of people in the Americas. In contrast to its close relative, *T. brucei* (see section 6), *T. cruzi* lacks an RNAi pathway. Hypotheses on this loss of RNAi pathways are well described in a review by Lon-Fye and colleagues [212]. A valuable observation that has been made from studying protozoan parasites, such as trypanosomes, is that there seems to be a direct relationship between the absence of mobile genetic elements and RNAi pathway deficiency. This is thought to be a direct result of the decay and finally the loss of function of these elements over an extended evolutionary period. The RNAi pathway therefore ceased to offer a selective advantage in these organisms and became lost without apparent consequences [1]. 

There are however four approaches to study *T. cruzi* that are noteworthy. Firstly, deep sequencing and genome-wide analyses revealed a significant number of short non-coding RNAs of which 79 do not show homology to known RNA classes. This study will provide insight into alternative RNA pathways in *T. cruzi* [213]. Secondly, there is research conducted to identify essential components of the RNAi pathway in *T. brucei* and to then transfer these to RNAi-deficient pathogens such as *T. cruzi,* enabling RNAi studies [22]. Thirdly, a recent study by Genovesio and colleagues used RNAi from the human host perspective to gain insight into the host-pathogen interface. By means of a genome-wide RNAi screen of human genes, they identified receptors as well as genes associated with calcium release and the TGF-beta signaling pathways, all involved in *T. cruzi* parasite entry. This study may pave the way towards novel entry-inhibiting drug targets [214]. Lastly, the development of integrative and episomal transformation allow for reverse genetic approached to be used, as well as rescue studies in *T. brucei* by means of *T. cruzi* equivalents [215]. 

## 6. RNAi in the Tsetse Fly and *Trypanosoma brucei*

### 6.1. RNAi Contributions to Understanding Vector Biology

Introduction of dsRNA into tsetse flies is done via feeding of flies on dsRNA containing bloodmeals or by *in vivo* injection [216]. To date a vast amount of genes have been silenced in the tsetse fly to gain insight into their biological functions and contribution to vector fitness. Some examples include silencing of Lipophorin, a lipid transport protein. Silencing of Lipophorin resulted in decreased lipid levels, delayed oocyte development and extended larval gestation periods but the findings still suggest another carrier lipoprotein to also be required for these processes in the tsetse fly [16]. Disruption of a nucleotidase-related protein in the salivary glands reduced anti-thrombotic activities by some 50%, confirming that nucleotidases in tsetse flies, similar to other haematophagous ectoparasites, constitute a major part of the vector’s anti-haemostatic machinery [17]. RNAi studies of two milk proteins GmmMGP2 and GmmMGP3, which are essential for viviparous reproduction, identified only GmmMGP2 as the gene impacting fecundity in tsetse flies. The discrimination power of RNAi revealed that only in GmmMGP2 knock-down flies, were ovulation, oocyte accumulation and degradation disrupted and fecundity reduced [217,218]. 

### 6.2. RNAi Contributions to Understanding Vector-Pathogen Interactions

Noteworthy is the number of papers in which RNAi was used successfully to disentangle host-pathogen interactions between tsetse flies and the causative agent of African and animal trypanosomiasis, *T. brucei*. In the past five years, the following insights have been gained: (1) Tsetse ALBA RNA-binding proteins are involved in trypanosome development *in vivo* [219]; (2) The EP protein is a potent immune-responsive molecule in the tsetse fly capable of reducing *T. brucei* infection in the vector midgut [15,220]; (3) Although 16 small Rab GTPases are present in *T. brucei*, three Rab proteins RabX1-3 are novel and play a role in fly infectivity [221]; (4)The bacterial endosymbiont of the tsetse fly, *Wigglesworthia glossinidiae*, actually protects the vector from being invaded by *T. brucei*. The interaction between the tsetse peptidoglycan recognition proteins, the bacteria, the level of anti-microbial peptides in the vector, and the mechanism of action are well described [222]. Finally, silencing of transferrin in the fat body tissues of tsetse flies increased the number of trypanosome infections, indicating a role for transferrin in protecting tsetse flies against infection [15].

One paper on understanding the host-vector interactions was published by Caljon and colleagues. In this study, tsetse flies were fed on rabbits to obtain anti-sera for subsequent screening of a cDNA expression library, resulting in the identification of tsetse antigen 5 (TAg5). In patients with allergic reactions to tsetse flies, IgE antibodies against TAg5 were detected, showing that this antigen is involved in the host-vector interface. Silencing of TAg5 in tsetse flies confirmed this observation as saliva from silenced flies displayed a reduced IgE binding potential. This study has contributed in confirming antigen-5 related proteins as functional allergens [223].

A standard PubMed search conducted with the terms *Trypanosoma brucei* and gene silencing or RNA interference indicate that 495 papers have been published using RNAi in *T. brucei*. We suggest reading some of the following reviews addressing new understanding of parasite biology and the identification of novel drug targets for parasite control [1,224,225,226].

## 7. RNAi: From Basic Research to Control Strategies

### 7.1. RNAi Contributes to the Identification of Candidate Transgenes

Transgenesis entails the transformation of vectors with transgenes that impair vector and/or pathogen development. This growing field of controlling vector-borne diseases is reviewed in [227,228,229]. Noteworthy is the contributions that RNAi-studies have made to the identification and evaluation of genes which are now used as transgenes to impact the mosquito vector-pathogen interactions (Table 3). Studies on transgenes affecting vector immune response genes such as Rel regulatory molecules (which affect the expression of numerous immune genes) and anti-microbial peptide genes such as defensins and cecropins, have pioneered this field [230]. With the growth in high-throughput screening studies, it is anticipated that the number of RNAi-discovered transgenes for vector-borne diseases will escalate within the next few years. 

**Table 3:** Mosquito transgenes disrupting mosquito-pathogen interactions. Adapted [227].

Vector Species	Transgene	Pathogen(s) targeted	Reference for RNAi of the transgene
*Mosquito immune response genes*			
*Ae. aegypti*	Defensin A	*Micrococcus luteus*	[231,232,233,234,235,236]
		*Plasmodium gallinaceum*	
		*Enterobacter cloacae*	
*Ae. aegypti*	Cecropin A	*Enterobacter cloacae*	
*An. gambiae*		*Plasmodium berghei*	
*Ae. aegypti*	REL-genes (REL1 and REL2)	*Plasmodium* spp.	
		*Bacillus subtilis*	
		*Escherichia coli*	
*Others*			
*An. stephensi*	SM1	*Plasmodium berghei*	[237]
*Ae. aegypti*	30Ka; 30Kb	Dengue Virus	[238]
*Ae. aegypti*	Anti-DENV2	Dengue Virus	[179,184]

### 7.2. Paratransgenesis

An additional strategy to reduce vector competence for disease transmission, involves genetic manipulation of natural endosymbionts of a particular vector species to produce molecules that impedes pathogen transmission or survival [239]. As described in this review, RNAi has already provided much insight into vector-pathogen interactions and paratrangenesis is already being used with success in vectors listed on VectorBase. This strategy was first described in *Rhodnius prolixus*, the Chagas disease vector, using its endosymbiont, *Rhodococcus rhodnii*. A gene encoding an antimicrobial peptide, cecropin A (which was evaluated using RNAi), was cloned into *R. rhodnii* that was stably expressed and released into the gut lumen, killing the disease causing pathogen *T. cruzi* with limited toxicity to the host tissues. Moreover, the transformed bacteria were stably maintained without the need for antibiotic selection and could be successfully transmitted to hosts with non-transformed endosymbionts due to the coprophagic habits of reduviids. In later studies, functionally active single chain antibody fragments (ScFv) were successfully expressed using a modified endosymbiotic *Corynebacterium spiecies* in the kissing bug, *Triatoma infestans*, as well as *R. rhodnii* in *R. prolixus* for control of *T. cruzi* transmission [239].

Other bacterial symbionts that have been successfully transformed and stably transmitted include *Sodalis glossinidius* (from Glossina sp.) and *Asia* sp. (from Anopheles spp.) [240,241]. Transformed Asia was also shown to be paternally transmitted in *An. stephensi*, providing the possibility of using non-hematophagous mosquitoes to spread modified endosymbionts for pathogen transmission control [242]. A reduction in vector competence may also be achieved by using genetically modified densovirusses in mosquitoes. In this regard, transmission of modified virus has been reported for *Ae. aegypti* and *A gambiae*, respectively [243,244]. To date, a number of endosymbionts have been identified may be used in paratransgenic control of insect-borne diseases from triatomine, tsetse fly and mosquito vectors [227]. However, few species have been successfully modified and shown to be stably transmitted to non-transformed individuals and their resultant progeny. In theory, genetically modified endosymbionts may not only provide researches with an additional tool for fighting disease but it can also deliver proteins and enzymes that test functional genetic hypotheses formulated from RNAi studies.

### 7.3. RNAi Together with Integrated High-Throughput Technologies Pave the Way for the Future in Combatting Pathogen and Their Vectors

The discovery of RNAi, for which Andrew Fire and Craig C. Mello were awarded the Nobel Prize in Physiology or Medicine in 2006, has once more demonstrated the importance of basic science in understanding molecular and physiological mechanisms. With this revelation the door was opened to the development of powerful approaches for investigation of functional genomics in a wide array of organisms, including those of importance in human and animal health. In the post genomic era, with a vast array of sequence information, RNAi provides a method to functionally annotate and subsequently validate the expanding spectrum of putative vaccine and drug targets, this is especially evident in the fields of tick and mosquito research.

RNAi in combination with recently developed technologies, such as high-throughput screening provides a platform to investigate proteins of vital importance in pathogen survival, transmission and the pathogen-vector-host interface. RNAi together with technologies such as deep sequencing revolutionized pathogen and vector research by enabling the comprehensive and refined annotation of the genomes of these organisms, leading to better knowledge of their RNA, proteins and metabolite derivatives. This finally provides an integrated systematic molecular biological approach for new optimized control strategies against these medical and veterinary important organisms [245].

Together with genomic data and RNAi integrated technologies basic biological questions regarding vectors and their pathogens are in the process of being elucidated. High-throughput sequencing provides an invaluable tool to furthermore investigate the full repertoire of RNAi machinery, to identify essential components of the pathway and even perhaps to reveal new classes of small silencing RNAs. This knowledge will allow for fundamental improvements in RNAi efficiency in RNAi-positive organisms and furthermore permit applied research, as the RNAi machinery can be reconstructed in organisms lacking the RNAi pathway, since the proof of concept has been confirmed in *Saccharomyces cerevisiae* [246].
